# Distribution and Blood Penetration of Hirudin in Various Organs and Tissues of Rabbits With Carotid Artery Injury by Ultraperformance Liquid Chromatography-Tandem Mass Spectrometry

**DOI:** 10.1155/jamc/5644566

**Published:** 2025-05-02

**Authors:** Yiran Feng, Lin Yang, Chunxia Guo, Ganyu Deng, Yudong Rao, Hao Zhou, Ying Zhang, Xueya Zhang

**Affiliations:** Department of Traditional Chinese Medicine, The First Affiliated Hospital of Kunming Medical University, Kunming 650000, Yunnan, China

**Keywords:** drug metabolism, drug uptake, hirudin, phenobarbital hirudin, ultraperformance liquid chromatography-tandem mass spectrometry

## Abstract

**Background:** Hirudin is a major active ingredient of the traditional Chinese medicine leech. It has been proved to have good antithrombotic and anticoagulant effects.

**Objective:** To determine the tissue distribution of hirudin and its pharmacokinetics in vivo by ultrahigh performance liquid chromatography-tandem mass spectrometry (UPLC-MS/MS).

**Methods:** A total of 21 SPF adult New Zealand rabbits were acclimatized for 1 week, and one was randomly selected as a blank control, while the remaining 18 were randomized to the control group and the model group. The heart, liver, spleen, lung, kidney, brain, plasma, blood vessels, and colon tissues were taken by execution at 1, 3, and 6 h. The samples were assayed using the natural hirudin standard as an external standard.

**Results:** The established UPLC-MS/MS method with good precision, accuracy, recovery, and stability of hirudin proved to be reliable. The correlation between different concentrations of natural hirudin standard and the response value was *R*^2^ ≥ 0.9997. The results of the distribution of various tissues showed that hirudin in the two groups had the highest content in plasma and blood vessels, followed by spleen, lung, and liver tissues, and was weaker in the brain and the heart, and the content of hirudin in the control group was higher than that of the model group, and the difference was statistically significant.

**Conclusion:** The absorption and metabolism of hirudin in rabbits with carotid artery injury mainly acted through vascular tissues and blood, and normal rabbits mainly metabolized it through spleen, lung, and liver.

## 1. Introduction

In recent years, percutaneous coronary intervention (PCI) has become a major therapeutic tool in the clinical treatment of coronary artery disease (CAD) [1]. Balloon catheters are medical devices used by PCIs to widen the blockage of atherosclerosis or vascular thrombosis, restore blood supply, and relieve downstream ischemic symptoms such as angina, myocardial infarction, and leg pain [2]. However, patients treated with PCI have carotid intimal hyperplasia or luminal stenosis due to vascular elastic retraction and platelet aggregation adhesion caused by balloon dilatation and stent placement [3, 4], could increase the probability of local or systemic thrombotic complications, inevitably leading to increased bleeding [5]. Therefore, adjunctive therapy with antithrombotic drugs after PCI plays a key role in patient recovery [6–8].

The traditional Chinese medicine leech has been recorded in “Shennong's Classic of the Materia Medica” in the Han Dynasty, claiming that it has the efficacy of removing blood stasis, expelling bad blood, and unblocking blood vessels [9]. It has been proved by modern scientific research that leeches have the effects of anticoagulation, antithrombosis, antiapoptosis, etc., and can be used in the treatment of various cardiovascular and cerebrovascular diseases [10]. Hirudin is a peptide isolated and extracted from the salivary glands of the Chinese medicine leech and is the active ingredient that plays a major role in the Chinese medicine leech [11]. Hirudin has a highly efficient and specific thrombin inhibiting effect, which can inhibit the formation of blood clots through direct target binding with thrombin [12]. Hu et al. found that hirudin could effectively reduce the formation of thrombus after femoral artery anastomosis in rats, thus improving the patency rate after vascular anastomosis [12, 13]. Su et al. [14, 15] found that hirudin reduced whole blood and plasma viscosity in hypercoagulable rats, demonstrating that hirudin has the ability to improve blood rheology and inhibit blood coagulation. However, no tissue distribution and pharmacokinetic studies of hirudin in carotid artery injury have been performed. However, no tissue distribution and pharmacokinetic studies of hirudin in carotid artery injury have been performed.

It is well known that the study of pharmacokinetics and tissue distribution is extremely important for drug development [16, 17]. He can assist in the prediction of various problems related to drug efficacy and toxicity [18, 19]. In order to understand the distribution of hirudin in the tissues of carotid artery-injured rabbits and to provide a basis for the clinical use of hirudin in the carotid artery injury, a simple, rapid, and sensitive ultrahigh performance liquid chromatography-tandem mass spectrometry (UPLC-MS/MS) method has been developed in the present study, and the distribution of hirudin, the main active ingredient of the hirudin, and its blood permeability have also been measured in 9 organs and tissues in carotid artery-injured rabbits after Fei Cow Hirudin's gavage treatment. The pharmacokinetics and distribution of hirudin in carotid artery injured rabbits were investigated.

## 2. Materials and Methods

### 2.1. Material

A total of 19 healthy adult clean-grade New Zealand White rabbits, male and female, weighing 2.5∼3 kg (Qingdao Kanda Aibo Biotechnology Co., Ltd.) were used, and all experiments were in accordance with the approval of the Animal Experimentation Center of Kunming Medical University (Approval no. kmmu2021069).

### 2.2. Reagents and Instruments

ACQUITY H-Class UPLC and XEVO-TQS triple quadrupole tandem mass spectrometer (Waters, USA) were used. Acetonitrile and formic acid (HPLC grade) and lyophilized powder of Fenugreek Hirudinea (0.3 g/strike, Lot no. C05-18081001) was purchased from Yunnan Hairidi Biopharmaceutical Co. Natural Hirudin Control (purity > 98%, Batch no.: 113274-56-9).

### 2.3. Animal Culture and Modeling

**Figure 1:**
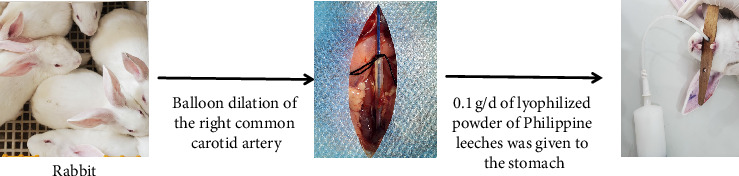
The balloon injury model of the carotid artery was established using New Zealand rabbits, and leech freeze-dried powder was administered to them by gavage.

Nineteen healthy adult New Zealand rabbits were cleaned, one was used as a blank control, and the rest were randomly divided into two groups, the control group, and the model group, with nine rabbits in each group. The model group was routinely reared 12 h before surgery. The right common carotid artery was balloon dilated. Rabbits were anesthetized with 1% isobarbital (5 mL-kg^−1^) injected intravenously from the ear, hair was removed, and the skin was disinfected with 75% alcohol. The right internal carotid artery, external carotid artery, and common carotid artery were isolated by exposing the trachea through a median jugular incision. A total of 100 U-kg^−1^ of heparin was injected intra-auricularly for anticoagulation, the external carotid artery was ligated at a distance of 0.5 cm from the bifurcation of the common carotid artery, and the common carotid artery was clipped proximally, the external jugular vein was incised, and the vein was punctured on the proximo-cardiac side of the ligature, and a balloon of 2.5 F in diameter was delivered to the common carotid artery at a distance of approximately 3 cm. Isotonic saline was injected into the balloon with a pressure pump, the pressure was maintained at 6∼8 kPa, and after filling the balloon, it was slowly dragged for 0.5∼1 min, with an interval of 1 min, and repeated for a total of 3 times so that the carotid artery was injured to a length of 3 cm, and the catheter was withdrawn, the external carotid artery was ligated, and it was sutured. Immediately postoperatively, penicillin injection 80,0000 U-d^−1^ was administered intraperitoneally. The control group did not undergo any surgery, and the Philadelphia leech lyophilized powder was administered by gavage 0.1 g-d^−1^ (diluted in a small amount of distilled water) (Figure 1). The safety assessment and acute toxicity testing of Philadelphia leech lyophilized powder is detailed in the Supporting Information (Supporting Report S1).

### 2.4. Sample Collection

New Zealand rabbits were modeled for 24 h after gavage of 0.1 g-d^−1^ lyophilized powder of pheonix leeches (diluted with a small amount of distilled water), and the experimental rabbits were killed at 1, 3, and 6 h after the administration of the drug, with three rabbits at each time point, and nine tissues of each rabbit were isolated and obtained from the blood plasma, brain, heart, liver, lungs, spleen, kidneys, large intestines, and carotid artery blood vessels.

### 2.5. Methods

#### 2.5.1. Preparation of Control Solution and Standard Curve Samples

An appropriate amount of natural hirudin control was weighed precisely and diluted with quantitative acetonitrile to prepare the standard working solutions of natural hirudin at the concentrations of 5.4, 54, 108, and 270 ng-mL^−1^ and then plotted on the standard curve. Using the external standard method for quantification, linear regression was performed with the substance content of hirudin as the horizontal coordinate and the peak area of the quantified ions as the vertical coordinate.

#### 2.5.2. Preparation of Test Solution

Accurately weigh 0.3 g each of plasma, brain, heart, liver, lung, spleen, kidney, colon, and carotid artery vascular tissues, add 1 mL of saline, homogenize on ice, centrifuge at 12000 r-min^−1^ for 10 min, take 100 μL of the supernatant, add 300 μL of methanol solution, mix with sufficient shaking, centrifuge at 12000 r-min^−1^ for 5 min, and take 5 μL of the sample for analysis.

#### 2.5.3. Chromatographic Conditions

A UPLC BEH C18 (100 × 2.1 mm, 1.7 μm) column was used; the temperature of the sample chamber was 10°C; the column temperature was 40°C; the flow rate was 0.4 mL-min^−1^; the mobile phase A was 0.1 formic acid 5 mmol-L^−1^ ammonium acetate solution; the mobile phase B was acetonitrile; and the gradient elution conditions were as follows: 0∼1 min, 10%∼90% acetonitrile; 1∼5 min, 10%∼90% acetonitrile; 5∼6 min, 50%∼50%; 6∼8 min, 10%. The gradient elution conditions: 0∼1 min, 10%∼90% acetonitrile; 1∼5 min, 10%∼90% acetonitrile; 5∼6 min, 50%∼50%; and 6∼8 min, 10%. The injection volume was 5 μL.

#### 2.5.4. Mass Spectrometry Conditions

Electrospray ionization (ESI) was used, ES+ was selected as the ionization mode, and the detection mode was multireaction static monitoring (MRM), capillary voltage: 3.20 KV. Ion source temperature: 150°C. Desolvent gas temperature: 350°C. Desolventization gas flow rate: 550 L-h^−1^. Cone pore gas flow rate: 150 L-h^−1^. Quantitative and qualitative analysis of hirudin was carried out using the MRM mode of monitoring ions m/z 1468.23 ⟶ 778.2 and m/z 1468.23 ⟶ 691.13.

#### 2.5.5. Accuracy, Precision, and Recovery Tests

A total of 100 μL each of intestinal, liver, kidney, heart, brain, spleen, lung, plasma, and vascular homogenate from rabbits in the blank group was diluted into 54, 108, and 270 ng-mL^−1^ of low, medium, and high concentration quality control samples. The samples were processed according to the method of “1.5.2” and then analyzed by UPLC-MS/MS.

#### 2.5.6. Stability Testing

The stability of hirudin was investigated by taking each tissue of blank rabbit. The stability of hirudin was investigated as the short-term stability at room temperature for 4 h, the stability of the injector for 24 h (at 4°C), the stability of repeated freezing and thawing for three times (at −20°C), and the long-term stability of the hirudin left for 7 days (at −20°C).

## 3. Results

### 3.1. Standard Curves and Quantification

**Figure 2:**
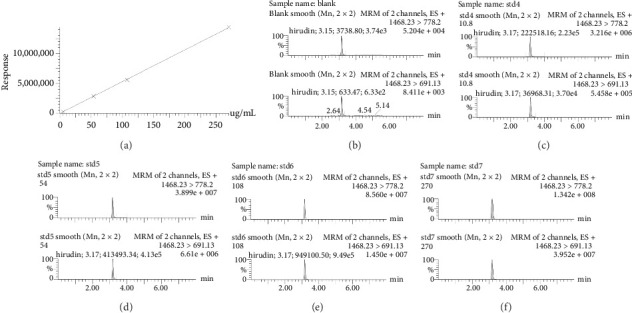
Chromatographic peaks and standard curves of the standards. Note: (a) Standard curve of natural hirudin standard; (b) chromatograms of blank plasma; and (c–f) chromatograms of natural hirudin standard 5.4, 54, 108, and 270 ng/mL.

Figure 2(a) represents the standard curve prepared with the response value as the vertical coordinate and the concentration of natural hirudin standard as the horizontal coordinate and obtained the regression equation *Y* = 53113*x* − 893.6, *R*^2^ = 1. It shows that hirudin has a good linearity in the concentration range of 5.4∼270 ng-mL^−1^, and the lower limit of quantification is 5.4 ng-mL^−1^. Figure 2(b) represents the chromatogram of the blank tissue homogenates, and Figures 2(c), 2(d), 2(e), and 2(f) represent the chromatograms of natural hirudin standards at 5.4, 54, 108, and 270 ng-mL^−1^. The peak time of blank plasma was 3.15 min, and the peak times of natural hirudin standard were 3.17, 3.17, 3.17, 3.16 min, respectively, which showed good separation and high instrumental response without obvious peak interference.

### 3.2. Examination of the Linearity of the Test Article

Pipette 100 μL of blank serum or blank tissue homogenate and then add the standard solution; hirudin standard is diluted to 54, 108, and 270 ng/mL series of concentration gradient, respectively, according to the “2.6 Preparation of test solution” under the method of processing samples, and then inject the sample. After that, the samples were injected into the sample and the linear relationship between peak area and concentration was calculated. Linear regression was performed with peak area (*Y*) as the vertical coordinate and concentration (*X*) as the horizontal coordinate, and the regression equations were calculated to obtain the standard curves and correlation coefficients of the three components, and the results are shown in Table 1.

### 3.3. Results of Precision Tests, Accuracy, and Recovery Measurements

The results of relative standard deviation (RSD) and relative error (RE) of the peak area of hirudin in serum and homogenate of heart, liver, spleen, lung, kidney, brain, colon, and vascular tissues of injured segments and the results of the spiked recoveries are shown in Table 2. The results showed that the intra- and interday precision (RSD) and the RE of hirudin were within 15%, which suggested that the precision of injection was good; the average recoveries of hirudin in serum and organ tissues were in the range of 95.06%∼98.09%, which was in accordance with the relevant regulations. The average recoveries of hirudin in serum and organ tissue homogenate ranged from 95.06% to 98.09%, which were in accordance with the relevant regulations.

### 3.4. Stability Test Results

The results in Table 3 showed that the precision and accuracy of the stability of hirudin under short term, repeated freezing and thawing, feeder, and long term were less than 15%, which proved that hirudin had good stability under different conditions.

### 3.5. Distribution of Hirudin in Various Tissues

The presence of hirudin was detected in the intestinal, spleen, lung, liver, kidney, heart, brain, plasma, and vascular tissues of rabbits in both groups. Hirudin concentration in all tissues of rabbits in both normal and model groups reached the maximum value at 3 h after the administration of Fenugreek hirudin jelly. Compared with the normal group, the concentration of hirudin in the tissues of the model group was significantly lower (*p* < 0.05), suggesting that carotid artery injury affects the uptake of hirudin in the spleen, lungs, liver, kidneys, and vascular tissues of the organism, see Table 4.

### 3.6. Blood Penetration of Hirudin

As shown in Table 5, the hirudin blood penetration rate was significantly different in spleen, lung, liver, kidney, and vascular tissues in both groups. The penetration rate in spleen, lung, liver, and kidney tissues was significantly lower than that in the control group, suggesting that carotid artery injury may affect the body's hirudin metabolism, but in the blood vessels of the injury group, the penetration rate of hirudin was significantly higher than that in the blood vessels of the control group, suggesting that hirudin may have the function of repairing blood vessels.

## 4. Discussion

Lipid-lowering and antithrombotic therapy remain the two cornerstones of pharmacologic treatment for AS. Yet, despite optimizing treatment strategies and lifestyle, ASCVD still carries residual risk. It is urgent and important to utilize the advantages of natural medicines of traditional Chinese medicine to reduce the risk of CVD ^[13]^. On the basis of the previous research on the protective effect of Fei Niu Hirudinea on the endothelium of AS-injured blood vessels, this group conducted a study on the distribution characteristics of hirudin, the main bioactive component of Fei Niu Hirudinea, in the carotid artery-injured rabbits, and its blood penetration rate, aiming to reveal the distribution target organs of Fei Niu Hirudinea, which is a potentially efficacious constituent of Fei Niu Hirudinea, and to provide a reference basis for the treatment of ASCVD. The purpose of this study is to reveal the target organs of the potential medicinal components of Fenu hirudin under the guidance of the theory of Chinese medicine “seeking the same qi and treating the same blood vessels” so as to provide a reference for the treatment of ASCVD.

Based on the basic theories of Chinese medicine, this group proposes a theory for the treatment of “thoracic paralysis and heart pain,” which covers the treatment of ASCVD diseases in modern medicine. Most of the insect herbs have mellow smell, compared with plant herbs, they are flesh and blood, sentient products, more easily absorbed and utilized by the human body, as the so-called “the same gas,” so the effect of special force. Because of this, the insect-based beverage preparations have been respected by medical practitioners throughout the ages. For example, the representative formulas created by the ancient medical sage Zhang Zhongjing, such as Rhubarb Euphrasia Pill, Turtle Shell Decoction Pill, Resistant Soup, and Lower Bruised Blood Soup, etc., are all made up of insect-based medicines. The theory of “treating blood and veins together” originates from the basic theory of Traditional Chinese Medicine (TCM), which believes that “the heart is the master of blood and veins,” meaning that the heart's qi pushes the blood to run in the veins and infuse into the whole body, playing the role of nourishment and moisturizing. When the heart is full of energy, the blood is full, and the veins are smooth, the heart will perform its normal physiological functions. “Blood disease” is mainly reflected in the abnormal contents of blood vessels, resulting in slow blood flow, stagnation, equivalent to hyperlipidemia, lumen wall thrombus, lumen thrombosis, and so on. The “pulse disease” is mainly reflected in the biological abnormality of blood vessel wall, which is equivalent to endothelial damage, plaque formation of blood vessel wall, lumen narrowing, and so on. The concept of “treating blood and veins together” emphasizes not only the need to remove phlegm and blood stasis by removing turbid and lowering lipids, i.e., the so-called “treating blood;” at the same time, it is also necessary to promote the repair of endothelial function by activating blood circulation and restoring vascular function, i.e., the so-called “treating veins.” This theory of “treating blood and veins at the same time” embodies the treatment concept of Chinese medicine's holistic view.

Most Chinese medicines are administered orally and are absorbed, metabolized and distributed to the tissues to exert their medicinal effects [20]. UPLC-MS/MS has the advantages of high sensitivity, short analysis time, high accuracy, and simple sample handling, which can accurately assess the distribution of hirudin in experimental rabbits. The hirudin used in this study was stable in acetonitrile solvent. The methodological investigation of several tissues showed that the precision, accuracy, recovery, and stability of hirudin in various tissues were all < 15%, which were within the acceptable range, indicating that the method was reliable. The results showed that the concentration of hirudin in all tissues reached a peak after 3 h, which proved that hirudin could be rapidly transferred to all tissues through the pathway of gastrointestinal tract-blood-tissue after the administration of Fenugreek leech to the experimental rabbits.

After instillation, the highest concentration of hirudin was found in plasma, indicating that hirudin could be rapidly absorbed by the blood, and the content in vascular, spleen, lung, and liver tissues was the next highest. In the two groups of experimental rabbit tissues, the concentration of hirudin in the vascular tissues of the model group was significantly higher than that of the control group, which proved that hirudin might repair the vascular injury induced by balloon placement. The difference in hirudin concentration in brain tissue was not significant, suggesting that hirudin is able to cross the blood–brain barrier and can be targeted for the treatment of cerebral infarction caused by carotid artery injury-induced thrombosis. Hirudin was metabolized by the liver, so the concentration of hirudin was higher in liver tissue after gavage administration, but the concentration of hirudin decreased with the metabolism of the liver as the time of administration increased. However, hirudin in the model group was significantly lower than that in the control group in spleen, lung, liver, and kidney tissues, which proved that hirudin in the carotid artery injury model group acted for a longer time in vivo than that in the control group. Also, the target distribution of hirudin in various organs will be affected by the body injury at the same time. However, due to the anticoagulant effect of hirudin, the uptake of hirudin in some tissues after injury was not significantly different from that in normal tissues. However, there are still some limitations in this study. Our current study does not include a comprehensive toxicological analysis of hirudin. However, the acute toxicity study report of Philadelphia leech freeze-dried powder (Supporting Report S1) indicates no significant acute toxicity in mice at the tested dosage. In contrast, its primary active ingredient, hirudin, may exhibit even lower toxicity. Although the application of hirudin in clinical practice appears to be safe and effective for now, conducting a comprehensive toxicological analysis of hirudin will contribute to a further evaluation of its safety.

In conclusion, UPLC-MS/MS can effectively detect the drug distribution of hirudin in various tissues of carotid artery-injured rabbits and provide a certain experimental basis for the targeted administration of hirudin for the treatment of PCI-induced vascular restenosis or local thrombotic complications.

## Figures and Tables

**Table 1:** Examination of the linearity of hirudin test samples.

Sample	Linear equation	*R*	Linear range (ng/mL)
Plasma	*Y* = 42709*X* + 555707	0.9818	50∼265
Heart	*Y* = 49183X − 75219	0.9952	50∼265
Liver	*Y* = 43883X − 24364	0.9939	50∼265
Spleen	*Y* = 38225X − 143871	0.9968	50∼265
Lung	*Y* = 34933*X* + 577987	0.9975	50∼265
Kidney	*Y* = 44240*X* + 218538	0.9989	50∼265
Brain	*Y* = 37566*X* + 595457	0.9827	50∼265
Colorectal	*Y* = 40751*X* + 170062	0.9978	50∼265
Vasculature	*Y* = 46557*X* + 3283.8	0.9864	50∼265

**Table 2:** The result of precision, accuracy, and recovery.

Sample	Concentrations (ng/mL)	Intra-day		Inter-day		Recovery (%)
		RSD (%)	RE (%)	RSD (%)	RE (%)	
Colorectal	54	5.1	−3.6	5.6	−1.2	96.36
	108	1.4	−1.5	5.5	−2.0	98.46
	270	3.2	−0.1	3.1	0.6	99.91

Spleen	54	6.4	−4.3	6.0	1.9	95.74
	108	4.4	0.0	2.6	−2.4	99.97
	270	3.3	−0.5	4.0	−5.4	99.53

Lung	54	4.7	−4.1	9.3	3.5	95.86
	108	7.1	−2.0	1.9	0.1	97.99
	270	2.7	−2.1	5.0	−3.6	97.94

Liver	54	4.7	−0.7	5.2	−1.4	99.26
	108	4.9	−4.5	6.2	−2.2	95.46
	270	1.4	−1.3	2.7	−1.4	98.72

Kidney	54	6.1	−3.6	10.1	−1.9	96.36
	108	3.1	−2.3	3.3	0.0	97.75
	270	3.3	−0.1	4.8	−2.7	99.93

Heart	54	4.3	−2.4	5.4	2.0	97.59
	108	3.0	−1.2	5.4	0.2	98.80
	270	5.4	−3.0	5.1	−2.4	97.01

Brain	54	2.8	−2.3	13.5	−5.5	97.65
	108	5.0	−1.8	3.2	1.4	98.21
	270	3.5	−0.6	2.2	−1.8	99.41

Plasma	54	5.9	−0.9	5.7	0.8	99.07
	108	4.4	−0.6	5.8	2.2	99.44
	270	3.1	−0.9	2.2	−1.3	99.07

Vasculature	54	3.6	−3.3	4.8	3.1	96.65
	108	6.3	−0.9	1.6	−0.1	99.10
	270	0.8	−1.0	3.8	−1.3	98.95

**Table 3:** Hirudin stability test results.

Sample	Concentration (ng/mL)	Short term (4 h)		Sample injector (24 h, 4°C)		Freeze/thaw (−20°C)		7 d (−20°C)	
		RSD (%)	RE (%)	RSD (%)	RE (%)	RSD (%)	RE (%)	RSD (%)	RE (%)
Colorectal	54	6.26	0.37	9.55	6.42	5.33	1.60	12.30	−0.37
	108	7.88	−2.16	3.85	−1.39	7.31	−1.67	6.55	0.31
	270	2.37	1.01	2.19	−0.14	1.14	1.80	4.63	−2.05

Spleen	54	10.90	−0.25	10.72	4.07	7.33	−6.42	12.32	−3.52
	108	3.36	−1.70	4.40	1.79	3.54	−0.90	8.59	−3.55
	270	3.73	0.98	2.36	1.06	4.05	−1.01	4.95	1.23

Lung	54	2.20	3.33	11.55	9.88	7.82	1.91	10.81	−0.86
	108	3.83	−0.37	3.32	−2.13	9.04	−2.25	6.24	−1.20
	270	2.69	−1.44	1.25	2.40	2.71	1.85	2.38	1.78

Liver	54	4.03	−3.15	4.21	6.67	8.15	2.59	13.34	−0.62
	108	4.15	−0.52	3.59	−0.62	7.09	0.52	9.31	−1.30
	270	1.11	1.69	2.02	0.99	3.83	0.43	3.98	−1.38

Kidney	54	5.82	2.41	6.60	0.37	8.22	−7.78	5.95	4.38
	108	3.70	−1.82	3.77	4.10	7.20	1.73	8.22	−4.91
	270	1.45	−0.04	3.25	−0.93	3.57	−2.05	3.38	0.21

Heart	54	9.66	0.37	3.89	−2.41	10.65	1.79	6.58	7.72
	108	2.31	−0.52	6.52	−2.93	2.63	−1.08	7.21	−2.38
	270	3.35	−0.89	3.35	−0.64	3.30	1.41	4.18	−1.49

Brain	54	6.59	1.17	10.77	4.01	6.42	−0.19	9.35	−1.48
	108	6.65	−2.93	7.29	−4.04	5.37	−2.53	2.17	1.88
	270	1.51	1.15	2.03	−0.94	1.96	0.93	3.35	−0.32

Plasma	54	6.41	1.91	5.05	4.27	9.83	3.81	5.20	6.91
	108	3.34	1.54	2.74	−3.70	5.13	0.99	3.24	0.33
	270	1.83	1.54	3.36	0.09	3.54	1.44	3.14	−0.10

Vasculature	54	5.53	2.10	5.99	−4.35	4.04	1.91	8.54	0.05
	108	2.53	−2.28	3.68	−4.10	2.62	2.95	5.52	2.25
	270	2.67	1.52	0.96	−2.59	3.06	1.82	3.23	−0.81

**Table 4:** Distribution of hirudin in various tissues of the rabbit.

Sample	Group	Concentrations (ng/mL)		
		1 h	3 h	6 h
Plasma	Control	194.2 ± 14	231.77 ± 10.45	193.07 ± 10.36
	Model	196.67 ± 10.13	237.17 ± 12.91	185.3 ± 9.01

Heart	Control	63.43 ± 7.84	78.8 ± 15.73	47 ± 3.94
	Model	48.57 ± 6.85	58.6 ± 3.87	44.37 ± 7.92

Liver	Control	164.83 ± 13.75	206.57 ± 18.1	175.03 ± 11.82
	Model	93.2 ± 9.52^∗^	109.6 ± 23.55^∗^	83.1 ± 6.62^∗^

Spleen	Control	150.87 ± 3.4	162.97 ± 9.43	108.93 ± 8.77
	Model	95.17 ± 8.4^∗^	113.63 ± 20.85^∗^	102.27 ± 4.24

Lung	Control	207.47 ± 75.32	176.03 ± 14.32	131.07 ± 3.17
	Model	93.2 ± 10.31^∗^	112.57 ± 12.95^∗^	79.63 ± 1.08^∗^

Kidney	Control	99.53 ± 7.84	109.37 ± 13.86	104.67 ± 7.44
	Model	57.87 ± 7.45^∗^	73.73 ± 6.3^∗^	56.63 ± 6.24^∗^

Brain	Control	19.37 ± 3.1	37.33 ± 4.32	24.5 ± 5.63
	Model	16.07 ± 3.95	27.17 ± 3.36	15.73 ± 2.32

Colorectal	Control	32.4 ± 4.3	50.69 ± 7.46	53.1 ± 7.18
	Model	11.9 ± 1.47	24.28 ± 4.84	27.48 ± 6.1^∗^

Vasculature	Control	90.5 ± 3.15	123.23 ± 15.31	85.9 ± 8.82
	Model	147.93 ± 2.89^∗^	184.77 ± 6.4^∗^	125.57 ± 13.04^∗^

*Note:* Compared with the control group, ^∗^*p* < 0.05.

**Table 5:** Blood penetration of hirudin.

Sample	Group	Concentration (ng/mL)	Penetrance rate (%)
Plasma	Control	231.77 ± 10.45	99.51 ± 4.49
	Model	237.17 ± 12.91	100.28 ± 5.46

Heart	Control	78.8 ± 15.73	33.83 ± 6.76
	Model	58.6 ± 3.87	24.78 ± 1.64

Liver	Control	206.57 ± 18.1	88.69 ± 7.77
	Model	109.6 ± 23.55	46.34 ± 9.96^∗^

Spleen	Control	162.97 ± 9.43	69.97 ± 4.05
	Model	113.63 ± 20.85	48.05 ± 8.82^∗^

Lung	Control	176.03 ± 14.32	75.58 ± 6.15
	Model	112.57 ± 12.95	47.6 ± 5.47^∗^

Kidney	Control	109.37 ± 13.86	46.96 ± 5.95
	Model	73.73 ± 6.3	31.18 ± 2.66^∗^

Brain	Control	37.33 ± 4.32	16.03 ± 1.85
	Model	27.17 ± 3.36	11.49 ± 1.42

Colorectal	Control	50.69 ± 7.46	21.76 ± 3.2
	Model	24.28 ± 4.84	10.27 ± 2.05

Vasculature	Control	123.23 ± 15.31	52.91 ± 6.57
	Model	184.77 ± 6.4	78.13 ± 2.71^∗^

*Note:* Compared with the control group, ^∗^*p* < 0.05.

## Data Availability

The data supporting the findings of this study are included within the article. Additional information can be obtained from the corresponding author upon reasonable request.

## Supporting Information

Supporting Report S1: Safety evaluation and acute toxicity testing report of freeze-dried powder of Philadelphia leeches.
