# Supplementary material for: Distribution and Blood Penetration of Hirudin in Various Organs and Tissues of Rabbits With Carotid Artery Injury by Ultraperformance Liquid Chromatography-Tandem Mass Spectrometry
Source: J Anal Methods Chem. 2025 May 2;2025:5644566. doi: 10.1155/jamc/5644566 (PMC12064311; doi:10.1155/jamc/5644566)
Supplement: Supporting Information — Additional supporting information can be found online in the Supporting Information section. [file 5644566.f1.zip › Supplementary Report S1/T1910001 English version.pdf]

Summary Report

Special Topic Name: Acute Toxicity Test of Hirudinaria manillensis Freeze-Dried Powder Administered Orally to Mice

Special Topic Code: T1910001

Name (Code) of the Test Article: Hirudinaria manillensis Freeze-Dried Powder

Test Article No.: TC1909001

Project Leader: Li Cong

Entrusting Unit: Yunnan Hairuidi Biopharmaceutical Co., Ltd.

Research Institution: Drug Safety Evaluation and Research Center, Institute of Medical Biology, Chinese Academy of Medical Sciences

Test Period: October 25, 2019 - November 1, 2019

Research Summary Report on "Acute Toxicity Test of Hirudinaria manillensis Freeze-Dried Powder Administered Orally to Mice"

Yunnan Hairuidi Biopharmaceutical Co., Ltd.:

The "Acute Toxicity Test of Hirudinaria manillensis Freeze-Dried Powder Administered Orally to Mice" entrusted by your unit has been completed. Now, the summary report is sent to your unit. Please check and accept it.

Attachment: "Research Report on Acute Toxicity Test of Hirudinaria manillensis Freeze-Dried Powder Administered Orally to Mice" (3 copies in total). The original (1 copy) is stored in the archives of the Drug Safety Evaluation and Research Center, Institute of Medical Biology, Chinese Academy of Medical Sciences.

Drug Safety Evaluation and Research Center, Institute of Medical Biology, Chinese Academy of Medical Sciences

Date: [Month] [Day], 2019

February

Table of Contents

- Confirmation and Approval of the General Report
- GLP Joint Statement
- Quality Assurance Statement
- General Information of the Research
- Research Content Abstract
- Results
- Conclusions
- Abnormal Situations Deviating from the Experimental Protocol
- References
- Appendix

Confirmation and Approval of the Summary Report

[Watermark]

[Signature Space]

GLP Joint Statement

Special Topic Name: Acute Toxicity Test of Hirudinaria manillensis Freeze-Dried Powder Administered Orally to Mice

Special Topic Code: T1910001

This test was completed in accordance with the requirements of the "Good Laboratory Practice for Non-Clinical Drug Studies" issued by the National Medical Products Administration. The research summary report truthfully reflects the test materials, methods, and results. There were no factors seriously affecting the test results during the test, and there were no deviations from the test protocol and standard operating procedures.

Signature of the Project Leader: Li Cong
Date: March 31, 2020

Quality Assurance Statement

Special Topic Name: Acute Toxicity Test of Hirudinaria manillensis Freeze-Dried Powder Administered Orally to Mice

Special Topic Code: T1910001

This project was inspected in accordance with the requirements of the "Good Laboratory Practice for Non-Clinical Drug Studies" implemented by the National Medical Products Administration. The test process, test protocol, relevant standard operating procedures, and original data are complete, and the test report is true and reliable.

Signature of the Head of the Quality Assurance Department: [Name]
Date: March 31, 2020

February

General Information of the Research

1. Special Topic Name, Special Topic Code, Research Objectives
   - 1.1 Special Topic Name: Acute Toxicity Test of Hirudinaria manillensis Freeze-Dried Powder Administered Orally to Mice
   - 1.2 Special Topic Code: T1910001
   - 1.3 Research Objectives: To observe the acute toxic reactions and mortality of ICR mice after single oral administration of Hirudinaria manillensis freeze - dried powder, reflect the direct damage of Hirudinaria manillensis freeze - dried powder to the body, and provide a reference for safe ranges in repeated dosing and clinical use.
2. Names and Addresses of the Research Unit and the Entrusting Unit

- 2.1 Non - Clinical Research Unit:
    - Name: Drug Safety Evaluation and Research Center, Institute of Medical Biology, Chinese Academy of Medical Sciences
    - Address: Huahongyuan, Xishan District, Kunming City
    - Postcode: 650108
    - Tel: 0871 - 68408567
- 2.2 Name, Address, and Contact Information of the Entrusting Unit:
    - Name: Yunnan Hairuidi Biopharmaceutical Co., Ltd.
    - Address: Huaxing Industrial Park, Youwang Town, Shidian County, Baoshan City, Yunnan Province
    - Postcode: 678200
    - Tel: 13708765007
- 2.3 Contact Person of the Entrusting Party:
    - Name: Luo Jiaojiao
    - Tel: 18087570139
    - Email: 1483368965@qq.com

3. Main Relevant Personnel
- 3.1 Project Leader:
    - Name: Li Cong
    - Tel: 1388492976
    - Email: k20qinkant.co.m
- 3.2 Responsible Persons and Participants for Each Research:
    - Preparation of Test Article: Zhou Lipeng, Wang Yixuan
    - Detection of Test Article: Du Tingfu, Wu Chengyang
    - Administration Personnel: Gao Jiahong, Li Cong
    - Observation, Weighing, and Food Intake Measurement: Zhou Lipeng, Gao Jiahong
    - Animal Quarantine and Feeding Management: Gao Jiahong, Li Yun
    - Gross Anatomy and Pathological Examination: Yang Jinling, Wu Chengping

4. Time Arrangement
- Animal Receiving Date: October 25, 2019
- Animal Marking Date: October 25, 2019
- Animal Quarantine Date: October 25 - October 28, 2019
- Animal Grouping Date: October 28, 2019
- Test Article Detection: October 23 - October 24, 2019
- Animal Administration Date: October 28, 2019
- Animal Dissection Date at the End of the Experiment: November 11, 2019
- Submission Date of the Summary Report to QA: November 18, 2019

5. GLP Regulatory Documents, Guidelines, and References Followed in the Research
- 5.1 Strictly implemented in accordance with the "Good Laboratory Practice for Non - Clinical Drug Studies" (GLP) issued by the National Medical Products Administration.
- 5.2 "Technical Guidelines for Single - Dose Toxicity Studies of Drugs" issued by the National Medical Products Administration.

6. Animal Use Management and Protection

The Drug Safety Evaluation and Research Center, Institute of Medical Biology, Chinese Academy of Medical Sciences has obtained the experimental animal use license. Before the implementation of this test protocol, it has been reviewed and approved by the Ethics Review Committee of the Institute of Medical Biology, Chinese Academy of Medical Sciences, with the approval number: DWSP201909003. The use of animals complies with the 3R (Reduction, Replacement, Refinement) principle. The relevant animal experiments are approved to be carried out.

7. Preservation of Original Records and Materials
   - Preservation Location: Central Archives
   - Preservation Period: 5 years after the new drug is launched on the market
   - Contact Person: Wang Yixuan
   - Contact Tel: 18288735065

Research Content Abstract

A single - gavage administration toxicity test was carried out using ICR mice. The acute toxic reactions and mortality of mice after single oral administration of batch - numbered 201906 - 1 Hirudinaria manillensis freeze - dried powder within 24 hours were observed. Forty healthy mice that had fasted for about 12 hours with free access to water were randomly divided into 2 groups, namely the negative control group and the Hirudinaria manillensis freeze - dried powder administration group, with 10 male and 10 female mice in each group.

The above two groups were respectively administered the control product and the test article once within 24 hours at the corresponding volume. On the day of administration, continuous observation was carried out for about 4 times. From the next day, cage - side observations were carried out once in the morning and once in the afternoon every day for 14 consecutive days. The main observations included various toxic reaction symptoms, signs, and mortality of the animals. The body weights were measured on the day of administration and on the 1st, 3rd, 7th, and 14th days after administration. After the observation period, the animals were grossly dissected, and the volume, color, texture, etc. of each organ were observed with the naked eye to check for any obvious abnormalities. The results showed:

1. General Symptoms, Signs, and Mortality
   - Negative control group: No abnormalities were observed in the animals.
   - Hirudinaria manillensis freeze - dried powder administration group: When administered at a volume of 40 ml and a test article concentration of 0.4 g/ml (the maximum prepared concentration), no abnormalities were observed in the animals.

2. Body Weight Situation

During the observation period, the average body weight and the average body weight gain of the animals at each time point were basically the same as those of the negative control group.

3. Gross Anatomy and Naked - Eye Observation of Animals

No animals died during the experiment.

At the end of the observation period, the surviving animals in the two groups were

grossly dissected and observed. No obvious abnormalities were found in the volume, color, texture, etc. of each organ.
Conclusion: Under the conditions of this test, when batch - numbered 201906 - 1 Hirudinaria manillensis freeze - dried powder was administered to ICR mice by gavage once within 24 hours, the maximum tolerated dose (MTD) was 16 g/kg, and no abnormalities were observed in the animals.

VII. Materials and Methods

1. Test Articles and Controls

1.1 Test Article

- Name: Freeze - Dried Powder of Hirudinaria manillensis
- Code: TC1909001
- Batch Number: 201906 - 1
- Appearance: Powder
- Content: 440U/g
- Expiry Date: 5 years
- Storage Conditions: Room temperature
- Storage Location: Sample Room
- Supplier: Yunnan Hairui Biopharmaceutical Co., Ltd.
- Reported Clinical Dosage: 1 - 3g per day per person

1.2 Negative Control

- Name: 0.9% Sodium Chloride Injection
- Batch Number: C190513H
- Specification: 250ml/bottle
- Expiry Date: April 2021
- Storage Conditions: Room temperature
- Storage Location: Sample Room
- Manufacturer: Kunming Nanjian Pharmaceutical Co., Ltd.

1.3 Preparation of Test Articles and Controls

- Weigh 20.000g of the Freeze - Dried Powder of Hirudinaria manillensis and put it into a 200ml beaker. Measure an appropriate amount of normal saline with a graduated cylinder and add it to the beaker containing the powder. Stir with a glass rod until well - mixed to prepare a 400mg/ml solution of the Freeze - Dried Powder of Hirudinaria manillensis. Then label the prepared solution.
- Prepare the solution fresh for use.

1.4 Testing of Test Articles

Refer to the test article testing analysis report.

2. Test System

2.1 Animals
- Name: ICR Mice
- Grade: SPF Level
- Quantity Purchased and Gender: 50 mice, 25 males and 25 females (including 10 reserve animals)
- Weight Range at Purchase: Males: 19 - 24.18g; Females: 19 - 20.9g
- Quantity Used and Gender: 40 mice, 20 males and 20 females
- Breeding Unit: Institute of Medical Biology, Chinese Academy of Medical Sciences
- Production License Number: SCXK (Dian) K2019 - 002

2.2 Reasons for Animal Selection
The ICR mouse strain has stable genetic traits and abundant background information. It is the most commonly used experimental animal in acute toxicity tests. This experimental system is widely used both at home and abroad, which is also beneficial for comparing the results with other experiments.

2.3 Animal Husbandry and Management
2.3.1 Feeding Cages
- Cage Type: High - Temperature - Resistant Polycarbonate (PC) Mouse Trays, with a volume of 300x200x130mm
- Stocking Density: 5 mice per cage
- Cage Replacement Frequency: Twice a week
- Methods for Handling Feces and Urine: Replace the bedding material when changing the mouse box
- Cleaning and Disinfection Methods: After changing the bedding material twice a week, use 0.1% Bromogeramine and 1% 84 Disinfectant alternately for disinfection. Rotate the types of disinfectants monthly.

2.3.2 Feed
- Name: SPF Mouse Feed
- Sterilization Method: Cobalt - 60 Radiation - Sterilized Pelleted Feed
- Manufacturer: Beijing KeXieLi Feed Co., Ltd.
- Address: No. 4, Yangshan Road, Chaoyang District, Beijing
- Production License Number: SCXK (Jing) 2019 - 0003
- Batch Number: 19093213
- Production Date: September 3, 2019
- Feeding Method: Ad Libitum
- Feed Storage: In the feed storage room of the barrier - environment animal facility, stored at room temperature.
- Quality Inspection and Analysis: Within the specified standard range, the feed supplier provides a test report for each batch of feed. The crude protein, crude fat, crude fiber, moisture, calcium, total phosphorus, and crude ash are tested in accordance with the standards of GB/T 6432 - 2018, GB/T 6433 - 2006, GB/T 6434 - 2006, GB/T 6435 - 2014, GB/T 6436 - 2013, GB/T 6437 - 2006, and GB/T 6438 - 2007. The compliance

is determined in accordance with GB 14924.3 - 2010, and the test report number is GNABSKGAISI088570. Lead, cadmium, mercury, BHC, DDT, and aflatoxin B1 are tested in accordance with GB 5009.11 - 2014, GB 5009.12 - 2017, GB 5009.15 - 2014, GB 5009.17 - 2014, and GB 5009.18 - 2016. The compliance is determined in accordance with GB 14924.2 - 2010, and the test report number is GNABSKGAIS1088571. The total number of colonies, coliforms, molds and yeasts, and the absence of Salmonella in the feed are tested in accordance with GB 4789.2 - 2016, GB 4789.3 - 2003, GB 4789.15 - 2016, and GB 4789.14 - 2016. The compliance is determined, and the test report number is GNABSKGATS1088572.

2.3.3 Drinking Water
- Name: High - Pressure Steam - Sterilized Water
- Sterilization Method: Sterilized at 121°C for 30 minutes before use
- Drinking Method: Ad Libitum
- Quality Inspection and Analysis: Sent to Yunnan Modern Ethnic Engineering Technology Research Center of Yunnan Academy of Science and Technology for testing. The test items include 30 physical and chemical indicators and 4 microbial indicators, all of which meet the requirements. The certificate number is YY201912 - 002G1.

2.3.4 Bedding Material
- Name: SPF Corncob Bedding
- Manufacturer: Beijing Ke'ao Xieli Feed Co., Ltd.
- Address: No. 4, Yangshan Road, Chaoyang District, Beijing
- Production License Number: SCXK (Jing) 2019 - 0003
- Batch Number: 19109811
- Production Date: October 8, 2019
- Storage: In the bedding storage room of the barrier - environment animal facility. The test report numbers are GPXGIS10A640PXKGAIS0A649.

2.3.5 Quarantine and Acclimatization
After receiving the animals, weigh all the animals in the quarantine room. The quarantine and acclimatization period is 3 days. During the quarantine period, the animals are fed with normal feed. Observe the animals daily after they enter the room. Waste should be hermetically packaged, autoclaved first, and then disposed of harmlessly. Cage equipment should be autoclaved for disinfection. Abnormal animals such as those with diarrhea, trauma, messy hair, or abnormal activities should be immediately culled. Animals diagnosed with infectious diseases should be immediately isolated, euthanized, and incinerated.

2.3.6 Animal Rearing Environmental Conditions
- Rearing Room: Barrier - Environment Animal Facility
- Temperature: 21.63±0.71°C
- Relative Humidity: 52.8±5.49%

- Lighting Time: 12 - hour light/dark cycle (lights on at 8 am - lights off at 8 pm, using an animal lighting control system)
- Air Exchange Rate: >15 times per hour
- Working Illumination: >200 Lx
- Animal Illumination: 15 - 20 Lx

2.4 Method for Individual Animal Identification

Mark the animals immediately after receiving them. Use ear - tagging for numbering. For male mice 1 - 25, the ear - tag numbers are 2201 - 2225; for female mice 1 - 25, the ear - tag numbers are 2226 - 2250. Clean the ear - tagging pliers with alcohol or disinfectant. Place the animal's ear on the ear - tag closing device, with the numbered side of the ear - tag against the pliers' jaws. Press the closing device tightly against the ear and clamp it into the cartilage. After clamping, make sure the tip of the ear - tag has passed through the hole.

2.5 Grouping and Group Identification

- Grouping Method: Group the animals by weight. For male mice: 1 mouse weighing 18 - 19g (including 1g), 2 mice weighing 19 - 20g (including 20g), 4 mice weighing 20 - 21g (including 21g), 8 mice weighing 21 - 22g (including 22g), 4 mice weighing 22 - 23g (including 23g), 3 mice weighing 23 - 24g (including 24g), and 3 mice weighing over 24g (cull 5 overweight mice). Then, distribute one mouse from each group into each experimental group in both forward and reverse order. For female mice: 1 mouse weighing 20 - 21g (including 21g), 10 mice weighing 21 - 22g (including 22g), 8 mice weighing 22 - 23g (including 23g), 6 mice weighing 23 - 24g (including 24g) (cull 5 overweight mice). Then, distribute one mouse from each group into each experimental group in both forward and reverse order.
- Number of Groups: Divide the animals into two groups, the experimental group and the negative control group, with 10 mice in each group. The weight difference of individual animals should not exceed 20% of the average weight. After grouping, identify the negative control group with a white cage card and the test article group with a red cage card.

2.6 Actual Rearing Method and Density of Animals during the Test, and Disposal Method for Remaining Animals

During the quarantine period, house 5 mice per cage. During the test, also house 5 mice per cage. Return the remaining animals after grouping to the animal husbandry and management department after the dosing is completed. If it is difficult to dose an animal on the dosing day or there is a need to replace an animal for other reasons, a mouse with a similar weight can be selected for replacement with the consent of the project leader.

3. Administration Route, Dose, Frequency, Treatment Duration and Rationale

3.1 Administration Route and Method

Administer the test article by gavage, which is consistent with the intended clinical route. Draw the control or test article solution into a syringe, insert the gavage needle

into the mouse's mouth, and slowly guide it along the back of the tongue and pharynx into the esophagus. After injection, withdraw the gavage needle.

3.2 Administration Frequency and Number of Doses

According to the requirements of the "Technical Guidelines for Single - Dose Toxicity Studies of Drugs" and combined with the characteristics of this test article, administer a single dose within 24 hours and observe the animals for 14 days.

3.3 Administration Method and Rationale

Fast the animals for about 12 hours before administration while allowing them free access to water. Administer the maximum dose by gavage, with the test article concentration at 0.4m (the maximum concentration). Set up a blank control group (0.9% sodium chloride injection) with 20 mice per group, housing male and female mice separately. The dosing volume for each group is 40ml/kg. To avoid false - positive reactions due to excessive fasting, resume normal feeding 30 minutes after administration.

3.4 Dosing Volume/Dose and Rationale

Clinical dosage: 1 - 3g per day per person. Administer the test article at the maximum concentration.

4. Observations and Measurements (Requirements for observing and measuring various indicators may vary depending on the test)

4.1 Observation of Clinical Symptoms

- Observation Frequency: Continuously observe the animals beside the cage until 4 hours after administration. From the next day, conduct cage - side observations once in the morning and once in the afternoon for 14 consecutive days.
- Observation Method: Observe all aspects of the test animals after administration, including their appearance, behavior, diet, secretions, excretions, manifestations and characteristics of poisoning, the time of onset and disappearance of toxic reactions, and the time of animal death. Record the corresponding symptoms and their severity in detail.

4.2 Body Weight Measurement

- Number of Measurements: Measure the body weight before grouping and dosing, and on days 1, 3, 7, and 14 after dosing.
- Number of Animals: All animals.
- Measurement Method: Weigh the animals in the morning.
- Instrument: YP20001 Balance.

4.3 Food Intake Measurement

- Number of Measurements: Measure the food intake once a week after dosing.
- Number of Animals: All surviving animals.
- Measurement Method: Provide sufficient feed for each animal every week. The

formula for calculating the food intake of each animal is: Food intake = (Amount provided - Amount remaining)/per mouse/per day.

4.4 Disposal of Dead and Moribund Animals and Pathological Examinations

During the test, if an animal dies or is moribund, perform a gross autopsy in a timely manner. Dissect the main organs such as the brain, heart, liver, lungs, spleen, kidneys, stomach, intestines, adrenal glands, thymus, testes, epididymis, ovaries, and uterus. For other animals, perform autopsies at the end of the observation period. Check if there are any changes in the volume, color, and texture of the organs. If so, fix the corresponding organs with 10% neutral formalin, embed them in paraffin, section them, and perform histopathological examinations under a microscope after HE staining.

4.5 Disposal of Surviving Animals at the End of the Test

Anesthetize and euthanize all animals with pentobarbital sodium. Perform gross autopsies and check for visible pathological changes in the main organs such as the brain, heart, liver, spleen, lungs, kidneys, stomach, intestines, adrenal glands, thymus, testes, epididymis, ovaries, and uterus. If there are any changes in the volume, color, or texture of the organs, fix the corresponding organs with 10% neutral formalin, embed them in paraffin, section them, and perform histopathological examinations under a microscope after HE staining.

VIII. Results

1. General Clinical Symptoms, Signs, and Mortality
   - Negative Control Group: No obvious abnormalities were observed in the animals in this group on the day of administration and during the observation period.
   - Freeze - Dried Powder of Hirudinaria manillensis Group: No obvious abnormalities were observed in the animals in this group on the day of administration and during the observation period.
2. Body Weight Changes
   - Negative Control Group: The body weights of the animals increased steadily at all stages.
   - Freeze - Dried Powder of Hirudinaria manillensis Group: The body weights of the animals increased steadily at all stages. During the observation period, the average body weight and the average body weight gain of the animals were basically comparable to those of the negative control group at each time point.
3. Food Intake Measurement
4. Mortality and Moribund Animals：No abnormal animal deaths occurred during the test.
5. Gross Autopsy：Perform gross autopsies on all animals. Observe the main organs including the brain, heart, liver, spleen, lungs, kidneys, stomach, intestines, adrenal glands, thymus, testes, epididymis, ovaries, and uterus with the naked eye. No obvious abnormalities were found in the volume, color, or texture of the animals' organs, so histopathological examinations were not performed.

IX. Conclusion

Based on the observations of clinical symptoms and mortality, the measurements of body weight and food intake, and the results of gross autopsies, under the conditions of this test, when the Freeze - Dried Powder of Hirudinaria manillensis (batch number 201906 - 1) was administered once to ICR mice within 24 hours, the maximum tolerated dose (MTD) was 6g/kg, and no abnormalities were observed in the animals.

1. Page 18 of 25
2. X. Deviations from the Test Protocol：This test was conducted in accordance with the requirements of the "Good Laboratory Practice for Non - Clinical Drug Studies" issued by the National Medical Products Administration. The research summary report truthfully reflects the test materials, methods, and results. There were no factors that seriously affected the test results, and no deviations from the test protocol and standard operating procedures.
3. Page 19 of 25
4. References：None
5. Page 20 of 25
6. Appendix 2：(Data Tables for Each Animal)
7. Page 21 of 23
8. Appendix 1
9. Page 22 of 25
10. Appendix 11：Animal Data Tables
11. Page 23 of 25
12. Page 24 of 25
13. Page 2
