# Supplementary material for: Distribution and Blood Penetration of Hirudin in Various Organs and Tissues of Rabbits With Carotid Artery Injury by Ultraperformance Liquid Chromatography-Tandem Mass Spectrometry
Source: J Anal Methods Chem. 2025 May 2;2025:5644566. doi: 10.1155/jamc/5644566 (PMC12064311; doi:10.1155/jamc/5644566)
Supplement: Supporting Information — Additional supporting information can be found online in the Supporting Information section. [file 5644566.f1.zip › Supplementary Report S1/T1910002 English version.pdf]

Summary Report

Subject Name: Repeated Dose Toxicity Test of Dry Powder of Hirudinaria manillensis by Gastric Administration in Rats
Subject Code: T1910002
Test Article Name (Code): Freeze - Dried Powder of Hirudinaria manillensis
Test Article No.: TCI909001
Subject Leader: Li Cong
Entrusting Unit: Yunnan Hairuidi Biopharmaceutical Co., Ltd.
Research Institution: Center for Drug Safety Evaluation and Research, Institute of Medical Biology, Chinese Academy of Medical Sciences
Test Duration: November 5, 2019 - January 6, 2026 (Safety Note)

Research Summary Report on "Repeated Dose Toxicity Test of Dry Powder of Hirudinaria manillensis by Gastric Administration in Rats"
Yunnan Hairuidi Biopharmaceutical Co., Ltd.,

The "Repeated Dose Toxicity Test of Freeze - Dried Powder of Hirudinaria manillensis by Gastric Administration in Rats" entrusted by your unit has been completed. Now, the summary report is sent to your unit. Please check and receive it.
Annex: "Research Report on Repeated Dose Toxicity Test of Dry Powder of Hirudinaria manillensis by Gastric Administration in Rats" (3 copies in total). The original copy (1 copy) is stored in the archives of the Center for Drug Safety Evaluation and Research, Institute of Medical Biology, Chinese Academy of Medical Sciences.

Center for Drug Safety Evaluation and Research, Institute of Medical Biology, Chinese Academy of Medical Sciences
Date: March 31, 2020

Table of Contents

- Confirmation and Approval of the Summary Report
- GLP Joint Statement
- General Information of the Research
- Results and Discussion
- Research Content Summary
- Materials and Methods
- Quality Assurance Statement
- Conclusions
- Abnormal Situations Deviating from the Experimental Protocol
- Appendices
- References

Research Content Summary

This experiment selected 120 adult Wistar rats, half male and half female. They were randomly divided into 4 groups (negative control group, low - dose group of freeze -

dried powder of Hirudinaria manillensis, medium - dose group of freeze - dried powder of Hirudinaria manillensis, high - dose group of freeze - dried powder of Hirudinaria manillensis) according to their body weights, with 10 rats in each group. The administration method was gavage. The rats were continuously administered with the freeze - dried powder of Hirudinaria manillensis (batch number 201906 - 1) for 4 weeks, and then had a 4 - week recovery period after drug withdrawal. The administration volume of each group was 15 mL/kg. The negative control group was given 0.9% sodium chloride injection. The low - dose group of freeze - dried powder of Hirudinaria manillensis had an administration concentration of 75 mg/mL, the medium - dose group had an administration concentration of 150 mg/mL, and the high - dose group had an administration concentration of 300 mg/mL of the suspension of freeze - dried powder of Hirudinaria manillensis.

During the test, the clinical symptoms of the animals were observed daily. The body weight and food intake were measured once a week. After the last administration and at the end of the recovery period, hematology, coagulation tests, serum biochemistry, ophthalmic examinations, and urine tests were carried out. After autopsy, bone smears were made, the wet weights of organs were measured, and tissue samples were collected for histopathological examinations.

The main inspection indicators are as follows:
1. Serum Biochemistry (15 items): Aspartate aminotransferase (AST), alanine aminotransferase (ALT), alkaline phosphatase (ALP), total bilirubin (TBIL), total protein (TP), albumin (ALB), blood urea nitrogen (BUN), creatinine (CREA), total cholesterol (CHOL), triglyceride (TGL), glucose (GLU), creatine kinase (CK), gamma - glutamyl transferase (GGT), potassium (K^+), sodium (Na^+), chloride (Cl^-).
2. Hematology Examinations: Red blood cell count (RBC), hematocrit (HCT), hemoglobin content (HGB/Hb), mean corpuscular volume (MCV), mean corpuscular hemoglobin (MCH), mean corpuscular hemoglobin concentration (MCHC), red blood cell distribution width (RDW), white blood cell count (WBC), platelet count (PLT), mean platelet volume (MPV), differential and percentage counts of white blood cells (LYM%, NEU%, BASO%, EOS%, MONO%), reticulocyte percentage (RETC%).
3. Coagulation Tests (4 items): Prothrombin time (PT), activated partial thromboplastin time (APTT), thrombin time (TT), fibrinogen (FIB).
4. Organ Weights: The wet weights of the brain, heart, liver, kidneys, adrenal glands, thymus, spleen, testes, epididymis, ovaries, uterus, and thyroid were measured, and the organ coefficients (wet weight of the organ g / body weight 100 g) were calculated.
5. Histopathological Examinations: Adrenal gland, aorta, bone (femur), bone marrow (thoracic spinal cord), brain (cerebrum, cerebellum, brainstem), cecum, colon, uterus and cervix, duodenum, epididymis (male), esophagus, eye, heart, jejunum, ileum, kidneys, liver, lungs (with main bronchi), lymph nodes (one related to the administration route and the other at a distant location), mammary gland, ovaries, fallopian tubes (female), pancreas, pituitary gland, prostate, rectum, sublingual gland, submandibular gland, gland, sternum, sciatic nerve, seminal vesicle, skeletal muscle,

skin, spinal cord (cervical vertebra, middle thoracic vertebra, lumbar vertebra), spleen, stomach, testes, thymus, thyroid, parathyroid gland, trachea, bladder, vagina, and all tissues with grossly observed abnormalities, tissue masses, and administration sites.

The above - observed indicators and measurement results were statistically analyzed respectively, and the results are as follows:
1. General Clinical Symptom Observation
   - Negative control group: No abnormal clinical symptoms were observed in the animals.
   - Freeze - Dried Powder of Hirudinaria manillensis Administration Groups: No abnormal clinical symptoms were observed in the low - dose, medium - dose, and high - dose groups.
2. Body Weight Measurement
   - During the administration observation period, compared with the negative control group, there were no significant changes with statistical significance in the average body weights of the low - dose, medium - dose, and high - dose groups.
3. Food Intake Measurement
   - Feed remained in the negative control group and the low - dose, medium - dose, and high - dose groups of the freeze - dried powder of Hirudinaria manillensis. In the first week of administration, there were significant differences between the low - dose, medium - dose, and high - dose groups and the negative control group. There were no differences among the administration groups. In the 3rd and 4th weeks of administration, there were significant differences between the high - dose group and the negative control group, and there were no differences between the low - dose and medium - dose groups. It is possible that the daily oral gavage of a certain volume of the suspension of freeze - dried powder of Hirudinaria manillensis affected the food intake, which is a normal situation. After drug withdrawal during the recovery period, there were no differences between the negative control group and the low - dose, medium - dose, and high - dose groups of the freeze - dried powder of Hirudinaria manillensis.
4. Serum Biochemistry
   - After the last administration, there were differences in the AST value between the low - dose group and the negative control group. There were significant differences between the medium - dose group and the negative control group, while there were no differences between the high - dose group and the negative control group. During the recovery period, there were differences between the medium - dose group and the negative control group. It is considered that there is no impact on serum biochemical indicators.
5. Hematology
   - After the last administration, the lymphocyte count (LY) in the medium - dose and high - dose groups had significant differences compared with the negative control group ($P < 0.01$), but there were no differences during the recovery period. There were no differences in other indicators, which is considered to have no toxicological significance.
6. Coagulation Tests

- After the last administration and 28 days after this administration, compared with the negative control group, there were no significant changes with statistical significance in the 4 coagulation indicators in the low - dose, medium - dose, and high - dose groups.

7. Ophthalmic Examinations

- Negative control group: No abnormal clinical symptoms were observed in the animals.
- Freeze - Dried Powder of Hirudinaria manillensis Administration Groups: No abnormal clinical symptoms were observed in the low - dose, medium - dose, and high - dose groups.

8. Urine Tests

- After the last administration and 28 days after the last administration, no abnormalities were observed in the negative control group and the low - dose, medium - dose, and high - dose groups.

9. Bone Marrow Count

- After the last administration and 28 days after the last administration, compared with the negative control group, there were no significant changes with statistical significance in the bone marrow cells in the low - dose, medium - dose, and high - dose groups.

10. Organ Weights

- After the last administration, there were significant differences in the uterine weight between the low - dose, medium - dose, and high - dose groups and the negative control group ($P < 0.01$), but there were no differences during the recovery period. 28 days after the last administration, only the ovarian weight in the high - dose group had a significant difference ($P < 0.01$). After the last administration, there were significant differences in the ovarian organ coefficients between the low - dose, medium - dose, and high - dose groups and the negative control group ($P < 0.01$), and there were differences in the uterine organ coefficients between the low - dose, medium - dose, and high - dose groups and the negative control group ($P < 0.05$), but there were no differences during the recovery period.

11. Pathological Examinations

- Gross Autopsy Results: No deaths or moribund animals occurred during the entire toxicity experiment.
- At the end of the administration period, the apparent pathological examinations of 80 Wistar rats showed no abnormalities. The results are shown in Table 2.
- At the end of the recovery period, the apparent pathological examinations of 40 Wistar rats showed no abnormalities. The results are shown in Table 3.
- Microscopic Examination Results
  - Heart: The histopathological examination results showed that at the end of the administration period, a. 1 rat in the control group and 1 rat in the high - dose group showed mild epicardial hemorrhage and a small number of inflammatory cells around the blood vessels or in the myocardium. At the end of the recovery period, a. 1 rat in the high - dose group showed blood vessel congestion and individual lymphocytes around it; b. 2 rats in the control group and 3 rats in the high - dose group had a small

number of scattered lymphocytes in the myocardium; c. 1 rat in the control group had individual pink protein cast - like structures locally; d. 1 rat in the high - dose group had mild sub - epicardial hemorrhage.

- Liver: The histopathological examination showed that at the end of the administration period, a. 2 rats in the control group and 2 rats in the high - dose group had a small amount of pink inflammatory exudate in the blood vessels, and 1 - 2 necrotic foci around or at the edge of the liver; b. 1 rat in the control group at the end of the administration period showed mild endothelial cell shedding; c. 2 rats in the control group and 4 rats in the high - dose group at the end of the administration period, and 4 rats in the control group and 2 rats in the high - dose group at the end of the recovery period had mild congestion in some blood vessels and a small number of inflammatory cells in the lumen.

- Lungs: The pathological results showed that the lungs of the rats in this project had varying degrees of hyperplasia, inflammatory cell infiltration, and a small amount of necrosis. Therefore, the pathological examinations of the lungs of the low - dose group and the medium - dose group in this project were supplemented. According to the extent of thickening of the pulmonary interstitium and the number of inflammatory cells, the lung lesions in this experiment were divided into 4 grades for scoring. The scoring criteria were: Grade 1: Mild (local) thickening of the pulmonary interstitium, with extremely few (scattered individual) inflammatory cells, denoted by "±"; Grade 2: Thickening of the pulmonary interstitium (partial), with a small number of inflammatory cell infiltrations, and may be accompanied by individual epithelial cell mass hyperplasia, denoted by "+"; Grade 3: The lesion shows a moderate inflammatory cell infiltration compared with Grade 2, and may be accompanied by individual (extremely few) fine - grained necrosis, denoted by "++"; Grade 4: The interstitium is thickened in a large - area sheet, with multiple inflammatory cell shedding and necrosis, a large number of inflammatory infiltrations, and may be accompanied by extremely few local parenchymal lesions of the pulmonary interstitium, denoted by "+++". The specific number and degree of lesions are shown in Table 5, the Summary Table of Pulmonary Histopathological Changes Scoring.

- In order to explore whether this pathological change is significantly related to the administration, the histopathological examinations of the lungs of all rats in the low - and medium - dose groups were carried out. The results showed that the main lesions of the lungs were different degrees of thickening of the pulmonary interstitium or increase of alveoli and inflammatory cell infiltration compared with those of the negative and high - dose groups.

- The histopathological examination results of the negative group and the high - dose group of the autopsy lung tissues showed that during the administration - period autopsy, there were 3 rats in the control group, 2 rats in the low - dose group, 2 rats in the medium - dose group, and 4 rats in the high - dose group, and during the recovery - period autopsy, there were 2 rats in the control group, 1 rat in the low - dose group, 2 rats in the medium - dose group, and 2 rats in the high - dose group. The main lesions of the lung tissues were mild bleeding in the lungs, with pink exudate in the blood vessels, local thickening of the interstitium, and a small number of inflammatory cells

(Grade 1); 11 rats in the control group, 10 rats in the medium - dose group, 8 rats in the high - dose group during the administration - period autopsy, and 4 rats in the low - dose group, 1 rat in the medium - dose group, and 1 rat in the high - dose group during the recovery - period autopsy had main lesions of lung bleeding, with inflammatory exudate, pink exudate in the blood vessels, partial thickening of the pulmonary interstitium, and a small amount of pulmonary type I epithelial cell hyperplasia around the blood vessels (Grade 2 lesions); 5 rats in the control group, 7 rats in the low - dose group, 6 rats in the medium - dose group, and 7 rats in the high - dose group during the administration - period autopsy, and 2 rats in the control group, 4 rats in the low - dose group, 1 rat in the medium - dose group, and 1 rat in the high - dose group during the recovery - period autopsy had main lesions of an increase in inflammatory cells in the lungs, with a small amount of pulmonary cell shedding and necrosis (Grade 3 lesions); 1 rat in the control group, 2 rats in the medium - dose group, 1 rat in the high - dose group during the administration - period autopsy, and 1 rat in each of the low - dose, medium - dose, and high - dose groups during the recovery - period autopsy had main lung lesions of severe lung bleeding, with large - area necrosis and shedding of alveolar septa, and diffuse infiltration of inflammatory cells around the lumen, on the lung wall, and in the alveolar septum (Grade 4 lesions).

- Pancreas: The histopathological examination showed that at the end of the administration period, 1 rat in the high - dose group had a small amount of pink exudate in some blood vessels.

- Adrenal Gland: The histopathological examination showed that at the end of the administration period, 1 rat in the control group had blood vessel congestion and scattered inflammatory cells.

- Kidneys: The histopathological examination showed that a. 1 rat in the high - dose group at the end of the administration period, and 2 rats in the control group and 2 rats in the high - dose group during the recovery period had a small number of glomerular contractions and an increase in the renal capsule space; b. 1 rat in the control group and 2 rats in the high - dose group at the end of the administration period had a small number of mildly increased glomerular capillaries; c. 2 rats in the control group and 2 rats in the high - dose group at the end of the administration period had blood vessel congestion and mild bleeding.

- Adrenal Gland: The histopathological examination showed that at the end of the administration period, 1 rat in the control group showed mild blood vessel congestion and a small number of inflammatory cells.

- Spleen: The histopathological examination showed that at the end of the administration period, 4 rats in the control group and 5 rats in the high - dose group, and 3 rats in the control group and 3 rats in the high - dose group during the recovery period all showed red - pulp area bleeding. Most of the lungs showed the deposition of brown - yellow - like granules.

- Testes: The histopathological examination showed that: a. At the end of the administration period, in 1 rat of the high - dose group, the structure of the seminiferous tubules in the testis collapsed, the structures of spermatogenic cells at all levels were unclear, some cells shed, and there were manifestations of necrosis such as nuclear

disappearance; b. During the recovery period, 1 rat in the control group showed a small number of bleeding points under the tunica; c. During the recovery period, 1 rat in the high - dose group showed mild bleeding and scattered small amounts of inflammatory cells.

- Ovaries and Fallopian Tubes: The histopathological examination showed that: a. During the recovery period, 2 rats in the control group showed mild ovarian bleeding and a small number of inflammatory cells; b. During the recovery period, 1 rat in the high - dose group had a small number of inflammatory cells in one lumen of the fallopian tube.
- Duodenum: The histopathological examination showed that: a. At the end of the administration period, 5 rats in the control group and 3 rats in the high - dose group, and 2 rats in both the control group and the high - dose group during the recovery period showed slight shedding of intestinal villi; b. Among them, 1 rat in the control group at the end of the administration period showed submucosal bleeding; c. At the end of the administration period, 1 rat in the high - dose group showed exfoliation of the intestinal villus epithelium and scattered small amounts of inflammatory cells.
- Jejunum: The histopathological examination showed that: a. At the end of the administration period, 3 rats in both the control group and the high - dose group, and 3 rats in the control group and 2 rats in the high - dose group during the recovery period showed slight shedding of the villus epithelium; b. During the recovery period, 1 rat in the control group showed submucosal bleeding.
- Ileum: The histopathological examination showed that: a. At the end of the administration period, 2 rats in the control group and 3 rats in the high - dose group, and 2 rats in the control group and 3 rats in the high - dose group during the recovery period showed slight shedding of the intestinal villus epithelium; b. During the recovery period, 1 rat in the control group had villus shedding and a small number of inflammatory cells.
- Colon: The histopathological examination showed that: at the end of the administration period, 1 rat in the high - dose group had exfoliation of the intestinal villus epithelium.
- Rectum: The histopathological examination showed that: at the end of the administration period, 1 rat in the high - dose group had congested submucosal blood vessels and a small number of lymphocytes.
- Related Lymph Nodes: The histopathological examination showed that: at the end of the administration period, 1 rat in both the control group and the high - dose group had lymph node bleeding.
- Pituitary Gland: The histopathological examination showed that: during the recovery period, 2 rats in the control group had scattered small amounts of inflammatory cells in the local area of the pituitary gland.
- Cervical Spinal Cord: The histopathological examination showed that: a. During the administration period, 1 rat in the control group had swelling of individual neurons in the gray matter area and disappearance of the nucleus; b. During the recovery period, 1 rat in the control group had scattered small amounts of inflammatory cells.
- Thyroid Gland: The histopathological examination showed that: during the recovery

period, 1 rat in each group of the thyroid gland had local thyroid gland follicular structure unclear and small - focal inflammatory cell infiltration; in the high - dose group of the thyroid gland during the recovery period, 1 rat had partial thyroid cell necrosis, unclear structure, and inflammatory cell infiltration.

- Bladder: The histopathological examination showed that: at the end of the administration period and the end of the recovery period, 1 rat in both the control group and the high - dose group had mild bladder bleeding.
- Skin: The histopathological examination showed that: at the end of the administration period, 1 rat in the high - dose group had dilated blood vessels in the dermis layer of the skin, and the lumen was uniformly red - stained.
- Trachea: The histopathological examination showed that: during the recovery period, 1 rat in the control group had a small number of red blood cells in the tracheal lumen and local mucosal shedding.

12. Situation of Dead Animals

No animal deaths were observed during the experiment.

13. Conclusions

Under the conditions of this experiment, the freeze - dried powder of Hirudinaria manillensis (batch number 201906 - 1) was administered by gavage. Rats were continuously administered for 4 weeks and then had a 4 - week recovery period after drug withdrawal. The administration volume of each group was 15 mL/kg. According to the body weights of the animals, the negative control group was given 0.9% sodium chloride injection, the low - dose group of the freeze - dried powder of Hirudinaria manillensis had an administration concentration of 75 mg/mL, the medium - dose group had an administration concentration of 150 mg/mL, and the high - dose group had an administration concentration of 300 mg/mL of the suspension of the freeze - dried powder of Hirudinaria manillensis.

During the test, compared with the negative control group, there were no differences in body weight, coagulation indicators, bone marrow cells, and general conditions in the low - dose, medium - dose, and high - dose groups of the freeze - dried powder of Hirudinaria manillensis. The differences in individual indicators of serum biochemistry, hematology, and organ coefficients were caused by accidental errors or individual differences. Histopathological examination: 1. The lungs of the rats in this project all showed varying degrees of lesions, but there was no obvious difference in dose and group distribution; 2. The lesions of other organs were all individual and occasional lesions, which had no obvious relationship with the drug.
